# P-593. Does Dolutegravir increase hypercholesterolemia and/or hypertriglyceridemia?

**DOI:** 10.1093/ofid/ofae631.791

**Published:** 2025-01-29

**Authors:** Zahid Talibi Alaoui, Malika Idalene

**Affiliations:** Mohammed VI University hospital, infectious disease department, Marrakech, Marrakech-Tensift-Al Haouz, Morocco; CHU Med VI, Marrakesh, Marrakech-Tensift-Al Haouz, Morocco

## Abstract

**Background:**

Antiretroviral therapy improved prognosis of people living with HIV (PLWA). WHO recommends Dolutegravir as the preferred treatment option for HIV in all populations considering its high genetic barrier and rare adverse effects compared to other treatments. In Morocco, the transition to Dolutegravir has begun in 2020 with the regimen: tenofovir desproxil fumarate/lamivudine/dolutegravir TDF/3TC/DTG (TLD). Some studies shown increased cholesterol and triglycerides levels on TLD regimens. The purpose of this study is to compare the effect of TLD on cholesterol and triglyceride levels compared to other antiretroviral therapy (ART) regimens.

**Methods:**

We conducted an observational comparative study at the Infectious diseases department Mohammed VI university hospital Marrakesh, MOROCCO. We included all PLWA admitted between 1st January 2021 and 31 December 2022 put on any ART and having a lipidic dosage. We excluded all patients with previous hypercholesterolemia and/or hypertriglyceridemia. We compared the occurrence of hypercholesterolemia and/or hypertriglyceridemia in PLHIV on TLD with those on other ART. For the statistical analysis a pearson chi-square test was used with the R software.

**Results:**

We included 173 PLWA; the median age was 33 years. 66.5% were male. The viral load was available in 152 people and was < 1000 copies/mL in 98%. The CD4 count was available in 161 persons and was >200 cells/mm3 in 69.6%. There were 120 persons on TLD arm (69.36%) and 53 on other ART arm (30.64%). On the TLD arm 15 people (12.5%) and 16 (13%) have developped an hypercholesterolemia and/or hypertriglyceridemia respectively versus 10 (18.86%) and 12 (22.64%) have developped an hypercholesterolemia and/or hypertriglyceridemia respectively on the others ART arm, but the difference was statistically non-significant (X2= 0.74; p=0.38) for hypercholesterolemia and (X2= 1.47; p=0.22) for hypertriglyceridemia.

**Conclusion:**

This study shows that there is no strong evidence of any association between the TLD regimen and the hypercholesterolemia and/or hypertriglyceridemia. The TLD regimen is more safe on the lipidic metabolism compared with other ART. Further large studies are needed to prove this benefit.

**Disclosures:**

**All Authors**: No reported disclosures

